# Creatine for Exercise and Sports Performance, with Recovery Considerations for Healthy Populations

**DOI:** 10.3390/nu13061915

**Published:** 2021-06-02

**Authors:** Benjamin Wax, Chad M. Kerksick, Andrew R. Jagim, Jerry J. Mayo, Brian C. Lyons, Richard B. Kreider

**Affiliations:** 1 Applied Physiology Laboratory, Department of Kinesiology, Mississippi State University, Mississippi State, MS 39759, USA; 2 Exercise & Performance Nutrition Laboratory, College of Science, Technology, and Health, Lindenwood University, St. Charles, MO 63301, USA; 3 Sports Medicine, Mayo Clinic Health System, La Crosse, WI 54601, USA; Jagim.Andrew@mayo.edu; 4 Department of Nutrition and Family Sciences, University of Central Arkansas, Conway, AR 72035, USA; jmayo@uca.edu; 5 Health, Kinesiology, and Sport Management Department, University of Wisconsin—Parkside, Kenosha, WI 53141, USA; lyons@uwp.edu; 6 Exercise & Sport Nutrition Lab, Human Clinical Research Facility, Department of Health & Kinesiology, Texas A&M University, College Station, TX 77843, USA; rbkreider@tamu.edu

**Keywords:** supplementation, ergogenic aid, athletic performance, weightlifting, resistance exercise, training, muscular power, recovery, muscular adaptation, muscle damage

## Abstract

Creatine is one of the most studied and popular ergogenic aids for athletes and recreational weightlifters seeking to improve sport and exercise performance, augment exercise training adaptations, and mitigate recovery time. Studies consistently reveal that creatine supplementation exerts positive ergogenic effects on single and multiple bouts of short-duration, high-intensity exercise activities, in addition to potentiating exercise training adaptations. In this respect, supplementation consistently demonstrates the ability to enlarge the pool of intracellular creatine, leading to an amplification of the cell’s ability to resynthesize adenosine triphosphate. This intracellular expansion is associated with several performance outcomes, including increases in maximal strength (low-speed strength), maximal work output, power production (high-speed strength), sprint performance, and fat-free mass. Additionally, creatine supplementation may speed up recovery time between bouts of intense exercise by mitigating muscle damage and promoting the faster recovery of lost force-production potential. Conversely, contradictory findings exist in the literature regarding the potential ergogenic benefits of creatine during intermittent and continuous endurance-type exercise, as well as in those athletic tasks where an increase in body mass may hinder enhanced performance. The purpose of this review was to summarize the existing literature surrounding the efficacy of creatine supplementation on exercise and sports performance, along with recovery factors in healthy populations.

## 1. Introduction

In the area of sports performance and exercise, both athletes and recreational non-athletes are continuously seeking competitive advantages to improve their health and optimize physical performance. Although various activities and considerations interact to achieve this end, many people turn to various exercise and nutritional strategies to augment performance (i.e., enhanced muscular strength, power, and force) [1,2]. One of the most commonly used and scientifically supported ergogenic aids is creatine monohydrate (commonly referred to as creatine) [1,3,4,5]. Creatine is an amino acid found in relatively high concentrations in skeletal muscle. Since 1992, when the first reports emerged that exogenous creatine monohydrate supplementation increases intramuscular phosphocreatine (PCr) stores [6], and shortly afterwards, when these increases were inextricably linked to augmented exercise performance [7,8], the ability of creatine to function as an ergogenic aid has attracted great interest. Still today, creatine is one of the most popular nutritional ergogenic aids for athletes and recreational performers [1,3,4]. In addition to its popularity in the consumer realm, creatine’s ability to enhance or augment some types of exercise performance has arguably been one of the most researched topics in the sport nutrition literature for the past 25 years [1,3,7,9,10,11]. In this regard, creatine has yielded predominantly positive effects regarding exercise performance measures with no ergolytic effects and minimal to no side effects in populations ranging from adolescents to the elderly [3,9]. The reported ergogenic benefits of creatine monohydrate include enhanced force output, augmented power output, increased strength, increased anaerobic threshold, increased work capacity, enhanced recovery, and enhanced training adaptations [1,3,4,9,12,13].

Although a complete discussion is beyond the scope of this review, several supplementation strategies have been explored to increase intramuscular creatine stores. A loading phase was initially proposed by Harris et al. in 1992 [6] and has subsequently been used in a large number of scientific investigations. This approach requires consuming four separate doses of 5 g/day for five consecutive days and consistently leads to a 20%–40% increase in creatine content [3]. Later, Hultman et al. [14] determined that smaller ‘maintenance’ doses (2–5 g per dose, 1 ×/day, or 0.03 g/kg/dose) could be used to maintain elevated creatine stores in the muscle. It is now commonly accepted that a loading phase may not be needed, but this approach remains the most rapid means to increase intramuscular PCr levels and, thereby, performance [14,15]. Notably, Law and colleagues [16] compared the efficacy of creatine loading on performance measures using a 2- and 5-day regimen (4 × 5 g/day) in 20 physically active men. They reported significant improvements in maximal leg strength and average anaerobic power following a 5-day creatine loading regimen compared to the placebo group; however, no significance in performance was found following 2 days of loading. Additionally, Sale et al. [17] found that the total ingestion of 20 g of creatine at 1 g per 30 min intervals for 5 days yielded lower urinary excretion of creatine than the typical loading regimen of 4 × 5 g/day over a 5-day period, leading the authors to conclude that this likely resulted in higher intramuscular levels. In this respect, it is without question that increasing intramuscular creatine stores through any number of supplemental approaches can increase intramuscular PCr levels and that these increases are directly linked to various ergogenic outcomes [3,9]. In this respect, Table 1 (adapted from: [3]) outlines the potential ergogenic benefits of creatine supplementation, whereas Table 2 provides examples of sports or sporting events that may be enhanced by creatine supplementation (also adapted from [3]). In addition to these tables, results from previous selected original investigations and review papers surrounding the ergogenic potential of creatine supplementation are summarized throughout this paper in tables. Finally, the interested reader is directed to other reviews that have outlined the impact of creatine supplementation on exercise performance [3,9,10,12,13,18]. The purpose of this review is to summarize the existing literature surrounding the efficacy of creatine supplementation on exercise and sports performance, along with recovery factors in healthy populations.

## 2. Materials and Methods

**Figure 1:**
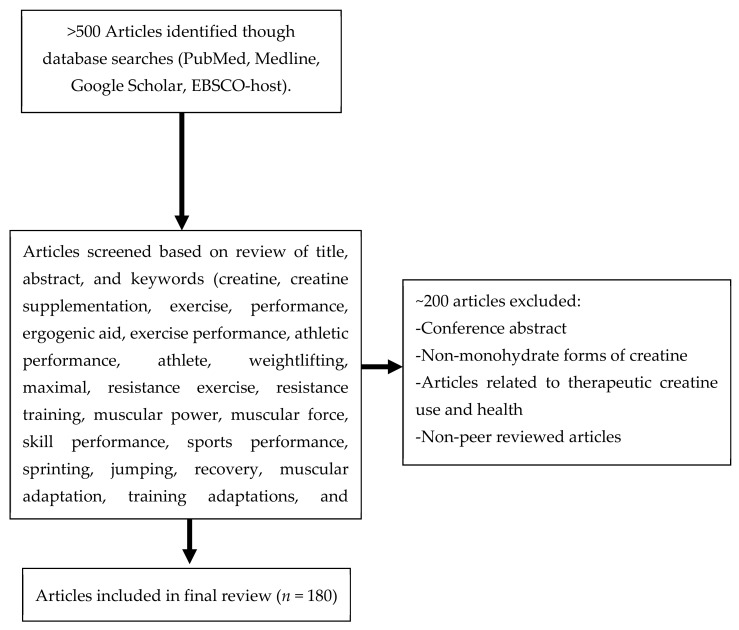
General flow diagram highlighting the selection process for included articles.

This review was completed using a narrative, non-systematic approach. A range of databases, including PubMed, Medline, Google Scholar, and EBSCO-host, were searched for this review paper (see Figure 1). A representative but non-exclusive list of keywords for these searches included: creatine, creatine supplementation, exercise, performance, ergogenic aid, exercise performance, athletic performance, athlete, weightlifting, maximal, resistance exercise, resistance training, muscular power, muscular force, skill performance, sports performance, sprinting, jumping, recovery, muscular adaptation, training adaptations, and muscle damage. Articles were chosen for inclusion based on the information they outlined and with a specific focus on exercise, performance, training adaptations, sport-specific skills, or recovery in healthy populations. Further citations were found, evaluated, and incorporated from the bibliographies of the selected literature. Articles with a focus on clinical applications or consisting of study protocols conducted among clinical populations were not included in the current review.

## 3. Exercise and Sports Performance

Creatine’s ability to increase various parameters of acute exercise performance is well documented [3,9,10]. A review by Kreider in 2003 summarized the literature and concluded that approximately 70% of these studies had reported an improvement in some aspect of exercise performance [9]. The magnitude of the increase in performance is dependent on a large number of variables, which can include the dosing regimen, training status of the athlete, and any one of a number of acute exercise variables (intensity of exercise, duration of effort, etc.). An overview of this literature reveals that performance increases of 10%–15% are typically observed [9,12]. More specifically, 5%–15% improvements in maximal power and strength, anaerobic capacity, and work performance during repetitive sprint performance are commonly reported, whereas improvements in single-effort sprint performance have been indicated to range from 1%–5% improvements. Alternatively, no consistent reports indicate that supplementation with creatine may have an ergolytic or performance-decreasing response. In this respect, a large number of studies have commonly reported an increase in body mass of 1–2 kg during the first week of loading [3], which may or may not have ergolytic implications, depending on the type of athlete and the phase of training. Finally, research involving various types of endurance activity in conjunction with creatine supplementation has received some attention as well.

### 3.1. Strength and Power

In most but not all studies, creatine supplementation has been demonstrated to be an effective ergogenic aid for increasing muscular strength and/or power, and these findings have included trained and untrained men and women, including athletes and non-athletes. The primary mechanism behind these ergogenic outcomes for creatine supplementation appears to be attributable, in part, to increases in intramuscular PCr concentrations [3,6,14,19]. Due to its potential not only to enhance strength and power output but also to expedite recovery from intense intermittent exercise, creatine supplementation has been shown to allow for increased volumes of work and increased work output during resistance training, which may then translate into greater strength gains [3,10,20,21].

#### 3.1.1. Short Term Benefits (<2 Weeks)

Improvements in strength and power performance have been observed following creatine supplementation. Generally, loading doses (i.e., 0.3 g/kg/day or ~20 g/day) are often used for short-duration supplementation periods or as part of a loading phase, which is commonly then followed by a more extended period of supplementation at a maintenance dose (i.e., 0.03 g/kg/day or 2–5 g/day). As intramuscular PCr stores increase through supplementation, subsequent improvements of 5%–15% in various performance parameters are commonly reported [10,22], sometimes even after relatively short periods of creatine supplementation (0–14 days). A study by Law et al. [16] reported significant improvements in average anaerobic power during a Wingate test and in back squat strength following a 5-day creatine supplementation protocol combined with a resistance training program. Interestingly, similar benefits were not observed after only 2 days of creatine loading, thereby indicating that perhaps >2 days of creatine loading is required to elicit significant strength and power-related benefits when combined with resistance training. Short-term benefits have also been reported in young adult males who were naïve to resistance training. After 10 days of creatine supplementation, improvements in the bench press and squat strength and power output were observed [23]. It is worth noting that subjects did not participate in a resistance training program during the supplementation period, yet ergogenic benefits were still present [23]. More recently, similar benefits in isometric leg strength were reported in young adult female futsal players following 7 days of creatine supplementation in conjunction with a concurrent resistance training program [24]. In contrast to these outcomes, creatine supplementation has not always yielded statistically significant ergogenic results. For example, following 7 days of creatine supplementation, maximal isometric knee extension strength was not altered in either the creatine or placebo group in 31 resistance-trained individuals who continued resistance training just as they had during the previous week [25]. Similarly, no strength differences were observed for 1 RM bench press or 1 RM leg extension exercises for either group after 7 days of creatine or placebo supplementation with no concurrent training in young adult males [26].

Thus, when attempting to glean meaning from numerous and varied study designs and supplementation protocols, it is crucial to look at how creatine was delivered, the nature of the subjects, whether or not concurrent resistance training occurred, whether the study design allowed for self-progression of training volume, the duration of the study, intended target outcomes, and several other factors. In this respect, a review by Rawson et al. [10] concluded that of the 22 identified studies on resistance training with creatine supplementation, the average increase in relative muscle strength (i.e., 1, 3, or 10 RM) was approximately 8% greater than that from resistance training alone. Furthermore, the average increase in muscular endurance (number of repetitions completed with a fixed load) was 14% greater than that following placebo ingestion and resistance training alone. More recently, a systematic review [18] identified 60 studies that met their criteria of randomized controlled trial study designs that utilized a double-blind, placebo control approach to examine the effects of creatine supplementation on lower limb performance. The outcomes of the review indicated an overall effect size of 0.336 and 0.297 for the strength increases in the back squat and leg press exercises, respectively. Furthermore, the calculated effect size for quadriceps strength was 0.266, with an overall global lower limb strength effect size of 0.266. Interestingly, these outcomes were observed independently of the target population or supplementation regimen, thereby supporting the overall efficacy of creatine supplementation, independently of outside-influencing factors. In a follow-up review, the same group [27] identified 53 studies utilizing similar search criteria but focused on the effects of creatine supplementation for upper limb strength. Results of the meta-analysis indicated an overall effect size of 0.265 and 0.677 for strength changes associated with the bench press and chest press exercises, respectively. For the pectoral exercise and global upper limb strength, effect sizes of 0.289 and 0.317 were found, again with no link with characteristics attributable to the population of supplementation regimen used, supporting the overall efficacy of creatine for upper body exercise performance independently of contextual factors. Table 3 provides a summary of selected studies that have examined the acute (<2 weeks) effects of creatine supplementation on strength and anaerobic performance.

#### 3.1.2. Longer-Term Training Adaptations (>2 Weeks)

Ergogenic benefits have also been reported following moderate-duration supplementation periods across several different populations. Although short-term benefits from creatine supplementation tend to be more specific to bouts of high-intensity exercise performance in regards to work output and anaerobic capacity, moderate-length supplementation periods appear to confer ergogenic benefits by helping to facilitate increases in training volume, thereby helping to augment training adaptations when combined with a structured training program. This indicates that consistent improvements in training quality and total work completed, underpinned by increasing intramuscular PCr content, are likely driving a lot of the training adaptations and benefits of creatine supplementation. These benefits appear to occur in both those who have been previously participating in resistance training programs and those who have been previously sedentary. For example, greater gains in 3 RM bench press were observed in experienced powerlifters supplementing with creatine compared to those supplementing with a placebo over a 26-day training period. Those in the creatine group also performed more total repetitions than the placebo group at 85% of 3 RM [28]. Similar results were reported in a study by Antonio et al. [29], who observed greater improvements in fat-free mass accrual and bench press 1 RM strength in recreational male body builders following a 5-week creatine supplementation period, in conjunction with a body-building-type resistance training program compared to those in the placebo group. Likewise, following a 6-week training period in experienced resistance-trained males, individuals supplementing with creatine displayed greater increases in bench press 1 RM compared to a placebo [30]. A 12-week concurrent periodized resistance training program paired with creatine supplementation resulted in significant improvements in bench press and back squat in 19 resistance-trained men. Additionally, improved jump squat performance was also observed. Interestingly, representing a protocol that is conducive to the performance-enhancing potential of creatine, subjects were provided with individualized training instruction throughout this study and were encouraged to progressively increase the intensity and volume of training throughout the study. The researchers noted that the creatine group displayed greater increases in exercise intensity and volume as the study progressed, which may partially explain the results [21]. In contrast, when subjects were involved in concurrent resistance training in which the training volume and intensity were controlled (i.e., subjects were prohibited from exceeding pre-determined numbers of repetitions and sets), it was concluded that creatine supplementation with concurrent weight training did not provide a greater ergogenic benefit when compared to the placebo group. This supports the notion that some of the ergogenic benefits are likely underpinned by facilitating increases in training intensity and work volume throughout a training program [31]. Table 4 provides a summary of selected studies that have examined the long-term (>2 weeks) effects of creatine monohydrate on strength and anaerobic performance.

#### 3.1.3. Athletes

From a historical perspective, some of the earliest work investigating the performance benefits of creatine supplementation was done as part of strength and conditioning programs in the late 1990s [22,32,33]. Several of these studies indicated that when creatine supplementation was provided for longer durations in conjunction with a strength and conditioning program, improvements in various indices of strength, power, and body composition were reported. These findings have since been replicated across a variety of sports, as seen in Table 4 and Table 5. For example, Kreider et al. [22] reported greater improvements in fat/bone-free mass, training volume, and sprint performance in NCAA Division I collegiate football players following 28 days of creatine supplementation in conjunction with a resistance/agility training program. Similarly, following 5 weeks of creatine supplementation, freshmen and redshirt American collegiate football players experienced greater increases in bench press and squat scores, and lower body power compared to a placebo [11]. Interestingly, in a similar study involving collegiate football players participating in an 8-week strength and conditioning program, athletes were provided wither creatine with dextrose at two different dosages according to each athlete’s fat-free mass (FFM) or a placebo for 8 weeks. Results revealed that both groups receiving creatine experienced significant strength gains over the training period. Still, only the creatine group (300 mg/kg) experienced significant strength gains compared to the placebo group. Thus, it appears that the dosage of creatine may be a factor when supplementing for some athletes [34]. Longer periods of creatine supplementation have also continued to show promise, with no risk of long-term benefits tapering off or individuals becoming “de-sensitized” to creatine’s beneficial effects. For example, Bemben et al. [35] observed superior improvements in the bench press and back squat 1 RM strength and anaerobic capacity in collegiate redshirt football players who had been supplementing with creatine compared to training alone. In a study involving collegiate football players participating in a 9-week resistance training program, results demonstrated improvements in the maximal bench press, squat, and power clean performances in all groups, with the creatine + glucose group evincing the greatest improvement compared to creatine alone and the placebo group. The authors suggested that this may have been due to increased work volume, facilitated by enhanced recovery [35]. Similarly, following a 10-week strength and conditioning program, collegiate football players who received low-dose creatine monohydrate supplementation (5 g/day) experienced greater increases in maximal bench press, squat, and power clean performances than from training alone. This study was important in that it revealed not only that creatine supplementation may improve strength and power, but that a loading phase was not necessary to achieve these results [36].

Analogous benefits have also been observed in female collegiate athletes supplementing with creatine in conjunction with a strength and conditioning program. In a study involving female collegiate lacrosse players participating in a concurrent resistance training program as part of a 5-week preseason conditioning program, those supplementing with creatine displayed even greater strength gains than the placebo group [37]. For example, during a 13-week resistance training program, collegiate female soccer players supplementing with creatine experienced more significant strength gains in the back squat between 5 and 13 weeks than those in the placebo group [38].

#### 3.1.4. Untrained

Creatine supplementation has even been shown to elicit ergogenic benefits to those who are previously untrained and in those who completed self-designed training programs. For example, improvements of 20%–25% in muscular strength have been reported in untrained females supplementing with creatine while undergoing 10 weeks of concurrent strength training [39]. Furthermore, Candow et al. [40] observed improvements in muscle thickness and leg press strength in men and women who were physically active but previously naïve to resistance training after 6 weeks of creatine supplementation and resistance training. Furthermore, 30 days of creatine supplementation (5 g/day) has also been shown to improve strength even without supervised concurrent strength and conditioning programs in individuals who are physically active [41].

### 3.2. Exercise Capacity/Prolonged High-Intensity Efforts

Harris et al. [6] were the first to show that supplementing with creatine in 4 × 5 g/day for 5 days resulted in a 50% increase in intramuscular total creatine content (20%–40% as PCr). Greenhaff and colleagues [8] translated these changes in total creatine content to performance outcomes when they had 12 study participants supplement with either a placebo or creatine over a 5-day supplementation period (4 × 5 g/day = 20 g/day) before completing five 30-s bouts of maximal muscle contractions. No performance changes were observed in the placebo group, but when creatine supplementation was employed, peak torque production increased in the final ten repetitions of the first bout and throughout the entirety of bouts 2–4. That same year, Balsom et al. [7] published data to illustrate the ergogenic potential of creatine supplementation. In this study, 16 healthy males supplemented for 6 days at a dose of 5 g/day in a double-blind format. Each participant completed 10 repeated 6-s bouts of high-intensity cycling with 30 s of rest between each bout. Work output exhibited smaller declines towards the end of each bout when creatine supplementation was provided; no such changes were observed in the placebo group. Birch and colleagues [42] supplemented individuals with either a placebo or creatine with 4 × 5 g/day (20 g/day) for 5 days. Before and after supplementation, study participants complete three 30-s bouts of high-intensity cycling. Although the placebo exhibited no impact on peak power, mean power or total work output, creatine supplementation significantly increased peak power after the first cycling bout and increased mean power output and total work completed during the first and second exercise bouts. No impact on power or total work was observed in the third bout. Casey et al. [43] had nine males complete two bouts of 30 s maximal cycling before and after supplementation with creatine at a dosage of 20 g/day for 5 days. Skeletal muscle biopsies confirmed an increase in intramuscular PCr content, which likely contributed to the increase in total work production of 4% that was observed. Moreover, the loss of ATP was found to be approximately 30% less when creatine was ingested, and this sustainment was found even after more work was completed. The results from this study were meaningful as they highlighted the key relationship that exists between the decay in exercise performance and intramuscular PCr status.

From there, modifications to the traditional creatine dose began to be considered, as studies had indicated some degree of individual variation (responders vs. non-responders) to supplementation [6,22,44]. As discussed previously, Kreider et al. in 1998 supplemented 25 football players for 28 days with a combination of carbohydrate (99 g) and 15.75 g of creatine and reported increases in fat-free mass, as well as improvements in bench press volume, total lifting volume, and total work performed through the first five sets of 6-s bouts of maximal cycling [22]. In accordance, Stout and investigators [45] examined the combination of creatine and carbohydrates. These authors reported that creatine supplementation (4 × 5 g/day for 6 days) in 26 college-aged men increased anaerobic working capacity by 9.4%, whereas no change in working capacity was noted when a 33-g dose of carbohydrate was delivered as a placebo. Notably, the combination of creatine and carbohydrates increased working capacity by 30.7%, providing additional credence to previous work that demonstrated an improvement in intramuscular creatine uptake and better sustainment of performance [6,43,44]. In 2003, Van Loon and colleagues [46] supplemented 20 college-aged men for 6 weeks in a double-blind, placebo-controlled, parallel fashion. A traditional loading phase (4 × 5 g/day for 5 days) was completed, which was then followed with 2 g/day for the remaining 37 days. Before and after the loading and maintenance supplementation periods, high-intensity exercise capacity was assessed by having participants perform 15 cycling sprints that were each 12 s in duration and separated by 48 s of rest. The loading phase successfully increased muscle creatine levels, which regressed back to baseline levels after 6 weeks. Additionally, peak power output after 5 days of supplementation increased performance during repeated sprints on the cycle ergometer. Moreover, these performance increases were maintained after the 6-week supplementation regimen, suggesting that continued ergogenic potential is exhibited by creatine under these parameters. Results from Barber et al. [47] illustrated in 13 healthy trained individuals that just 2 days of supplementation in a randomized, double-blind, crossover fashion with either a placebo (20 g maltodextrin + 0.5 g/kg maltodextrin) or creatine (20 g + 0.5 g/kg maltodextrin) prior to completing six repeated 10-s cycling sprints with 60 s of rest between sprints can increase relative peak power production by 4% when compared to the placebo condition. Moreover, Fukuda and colleagues reported that acute creatine supplementation significantly increased anaerobic running capacity, an intense, challenging three-minute bout of high-intensity running [48]. In summary and when viewed collectively, these results and others display a consistent pattern of research that supports the ability of creatine supplementation to increase one’s capacity to perform high-intensity exercise both after acute (5–6 days) and prolonged (4–6 weeks) periods of supplementation.

### 3.3. Sport-Specific Performance

For many athletes and coaches, the impact of creatine supplementation on sports performance is the most important consideration. It is well established that creatine supplementation leads to increased muscle PCr content [6,14], accelerated ATP resynthesis [7,43], and enhanced performance in short-duration, predominately anaerobic intermittent exercise [13,49]. As a result of these observed benefits, it has been suggested that creatine supplementation could translate to enhanced on-field performance for competitive athletes. The ever-changing nature of sports in terms of intensity, distances covered, and duration makes the replication of sports performance difficult. Due to the challenges associated with assessing on-field performance, many experimental approaches have employed simulated play and field tests. The outcomes and designs of several studies have been summarized in Table 5.

#### 3.3.1. Agility Performance

Numerous studies have measured performance using sport-specific drills and simulations following creatine use. Assessing agility performance is a common way to assess on-field performance during soccer. In this respect, Cox et al. [50] had 14 elite female soccer players undertake a simulated match before and after consuming either 20 g/day of creatine or placebo for 6 days. During the simulation, performance times during ten agility runs were digitally recorded and run times were significantly faster during trials 3, 5, and 8 in the creatine supplementation group. Similarly, Ramirez-Campillo et al. [51] divided 30 competitive female soccer players into three equal groups (creatine, placebo, control) with each completing a pre-test agility run. The creatine and placebo groups were also assigned to a 6-week plyometric training program. Although agility run times increased in both groups as a result of training, no performance differences between groups were identified. Other soccer studies have examined field tests, with mixed results. For example, Ostojic et al. [52] had 20 adolescent male soccer players ingest 30 g/day of creatine for 7 days, which resulted in an improvement in ball dribbling skills, jump height, and power production, whereas no improvement in kicking accuracy was observed. Similar outcomes in kicking accuracy were reported by Cox et al. [50] after creatine supplementation.

Tennis is a sport characterized by explosive, powerful actions interspersed with tremendous agility demands and notable exercise capacity demands. However, the impact of creatine ingestion on tennis performance has not been as extensively studied as other sports. Using a double-blind cross-over study design, Op’t Eijnde et al. (2001) had eight well-trained tennis players consume 20 g/day of creatine or a placebo over 5 days. Stroke quality via the Leuven Tennis Performance Test and a 70-m shuttle run were measured at baseline and after each treatment [53]. The study included a 5-week washout period between treatments and no significant differences in performance were recorded after creatine supplementation for either test. Pluim et al. (2006) had 39 male tennis players perform ball machine ground stroke drills to assess the acute (0.3 g/kg/day for 6 days) and chronic (0.03 g/kg/day for 5 weeks) effects of creatine ingestion [54]. Performance metrics included the velocity of repeated ground strokes and serving velocity. No significant differences were found in these performance measures after the acute loading phase or following 5 weeks of creatine use in any performance measure. As a result, the authors concluded that creatine should not be recommended to tennis players; however, the chosen outcome measures may not have been ideal outcome variables when considering creatine’s primary ergogenic mechanism of action.

Studies that have incorporated creatine use in combat sports have yielded conflicting results. Kocak and Karli (2003) had 20 international-level wrestlers perform a 30-s Wingate test before and after consuming 20 g/day of creatine or placebo for 5 days. Average power and peak power were significantly greater when creatine supplementation was provided, whereas no change was observed in the placebo condition [55]. However, Aedma et al. [56] reported creatine supplementation (0.3 g/kg/day) for 5 days did not improve peak power, mean power, or fatigue index in repeated upper-body ergometer sprint tests in 20 amateur Brazilian Jiu Jitsu and submission wrestling athletes. Similarly, 10 taekwondo athletes failed to show improvements in Wingate anaerobic capacity tests following the use of creatine (50 mg/kg/day) or placebo for 6 weeks in a crossover design with a 6-week washout period [57]. In summary, the available research in combative sport athletes suggests that creatine supplementation may have limited potential to enhance performance in these types of sports. These results are somewhat surprising, considering the energetic nature of these events, and as a result, more research is encouraged to better determine the potential for creatine to enhance performance in sports of this nature.

#### 3.3.2. Sprint Performance

Improvements in sprint performance after creatine supplementation have been shown in handball [58], football [59], ice hockey [60], soccer [50,51,52,61], swimming [62], and track athletes [63]. Briefly, Kreider in 1998 [22] and Stout in 1999 [59] reported improvements in sprint performance by collegiate American football players after supplementing their diet with combinations of creatine (5.25–15.75 g) and carbohydrates (33–99 g). Similarly, Skare et al. [63] reported faster 100-m sprint times and reduced total time of 6 × 60-m sprints in a group of well-trained male sprinters after 5 days of creatine supplementation (20 g/day). Not all studies, however, suggested that creatine supplementation improves sprint performance [54,64,65,66]. In this respect, Glaister and colleagues [67] reported no change in sprint running performance (30 m dash) after a standard 5-day creatine loading phase in active college-aged men who were consistently completing activities that involved sprinting. Similarly, Delecluse et al. [64] also reported that creatine supplementation did not improve 40-m dash performance after a 7-day creatine loading protocol in elite college sprinters. It was surmised from these authors that the high volume of high-intensity training already being completed by these athletes may have underpinned creatine’s ability to promote further improvements in sprinting performance. This thesis, however, is refuted by Skare and investigators [63], who supplemented collegiate track and field athletes with creatine for five days (20 g/day) while also participating in a concomitant resistance training program. In this study, performance changes in 60-m and 100-m sprint performance were assessed and the authors found that 100-m sprint velocity was improved and the total time to complete multiple 60-m sprints was significantly reduced. In summary, it appears that creatine’s ability to improve performance in those activities which contain a predominant gravitational component (e.g., sprinting, vertical jump, etc.) may be challenged. Thus, it has been suggested that the added body mass which occurs due to creatine supplementation may offset any ergogenic outcomes, but this conclusion has not been universally observed. As a result, people must consider that multiple factors interact (training status, type of athlete, sprint distance, the incorporation of other forms of training besides sprinting, etc.) to influence the final observed outcomes. Nevertheless, a number of studies are available that have documented the ability of creatine supplementation to improve sprinting performance.

#### 3.3.3. Jump Performance

The ability to jump explosively both horizontally and vertically is a key attribute for many athletes. To jump effectively, an athlete must be able to generate high amounts of power relative to their body mass. Due to creatine’s known ability to increase body water and subsequently body mass, concerns have been raised about the impact of supplementing with creatine on jumping performance. Lamontage-Lacasse et al. [68] compared the effect of 4 weeks of creatine to that of a placebo on 1 RM spike jump (SJ) and repeated block jump (BJ) capacity among 12 elite male volleyball players. Dosing for the study included ingesting either a placebo or creatine at 20 g/day on days 1–4, 10 g/day on days 5–6, and 5 g/day on days 7–28. Before and after the treatment, subjects performed the 1 RM SJ test, followed by the repeated BJ test (10 × 10 BJs). Following supplementation, no differences were observed in SJ between the creatine and placebo conditions. A non-significant (*p* > 0.05) 1.9% improvement in BJ performance was observed for the group supplementing with creatine compared to the placebo group. Alternatively, Izquierdo et al. [58] found that creatine supplementation (20 g/day for 5 days) attenuated a decline in countermovement jumping (CMJ) ability after a single set of half squats when compared to the placebo group. Similar findings were recorded by Mujika et al. [61], who found that 5 days of creatine loading (20 g/day) limited the observed decay in jumping performance after a maximal intermittent soccer-specific test (40 × 15-s bouts of high-intensity running interspersed with 10-s bouts of low-intensity running). Additionally, a recent meta-analysis in soccer suggested that creatine supplementation produces small but non-significant improvements in single jump performance [69]. Stone et al. [11] supplemented 42 American collegiate football players for 5 weeks and determined that creatine supplementation led to significantly greater power output and rate of force development during static vertical jumps. In addition, Haff et al. [70] reported greater rates of improvement in CMJ performance for track athletes who ingested creatine (0.3 g/kg/day over the 6-week study) compared with a placebo group. To summarize the available literature surrounding jumping performance, the majority of studies demonstrate consistent, small improvements in jumping performance, whereas other studies have reported improvements that did not reach traditional levels of statistical significance.

#### 3.3.4. Selected Competitive Athletes

##### American Football

Using trained collegiate athletes, Kreider et al. [22] had 25 NCAA Division IA football players supplement in a double-blind, randomized fashion for 28 days with either 99 g of carbohydrates or an isoenergetic amount of carbohydrate + 15.75 g of creatine as part of their regular training and conditioning sessions. Increases in body mass and fat-free mass were found, along with increases in bench press volume, total volume, and total work completed during the initial bouts of a repeated sprint cycling protocol. In addition, Stout and investigators [59] supplemented 24 collegiate football players with either carbohydrates (35 g), creatine (5.25 g of creatine + 1 g of carbohydrates) or creatine + carbohydrates (5.25 g of creatine + 33 g of carbohydrates). Four doses were taken per day in all groups for the first 5 days, whereas thereafter, two doses per day were consumed. The combination of creatine + carbohydrates led to significantly greater improvements in bench press strength, vertical jump, and 100-yard dash when compared to the carbohydrate group, whereas the observed changes in the creatine only group exhibited greater mean changes, but the magnitude of these changes was not considered to be statistically significant. Stone and colleagues [11] reported increases in upper and lower body strength and peak rates of force development in American football players after 5 weeks of creatine supplementation. Finally, Kreider in 1999 [33] supplemented 51 college football players in a matched, randomized fashion to a placebo, carbohydrates + placebo, or a combination of carbohydrates + protein + creatine. The two groups which contained creatine experienced the greatest gains in lean mass in comparison to the groups that did not contain creatine.

##### Track and Field

Kirksey et al. [71] supplemented 36 male and female collegiate track and field athletes with either a placebo or creatine (0.30 g/kg/day) for 6 weeks. Lower body power was assessed using countermovement and static vertical jumps, whereas exercise capacity was assessed using five consecutive 10-s bouts of cycling exercise. Improvements in countermovement vertical jump height (7.0% vs. 2.3%), vertical jump power (6.8% vs. 3.1%), average cycling peak power (12.8% vs. 4.8%), average cycling power (10.8% vs. 3.1%), and cycling total work (10.8% vs. 3.5%) were all greater when athletes supplemented with creatine vs. placebo. To further extend these outcomes, Lehmkul et al. [72] supplemented 29 male and female collegiate track and field athletes for 8 weeks and assessed changes in body composition and performance. Again, supplementation with creatine after 1 week and also after an additional 7 weeks resulted in improvements in rates of power production when compared to rates observed by athletes who consumed a placebo.

##### Swimming

Grindstaff and investigators [73] supplemented 18 male and female junior competitive swimmers with either creatine or a placebo for 9 days during training. Before and after supplementation, all swimmers completed three 100-m freestyle swims with 60 s of recovery between each race. Those swimmers supplementing with creatine swam significantly faster than those supplementing with a placebo after the first heat. The entire distance covered by those supplementing with creatine over all three 100-m races tended to be greater. This study is significant as it was one of the first to use adolescent athletes and also incorporated female athletes. Additionally, Peyrebrune et al. [65] reported improvements in repeat swim performance among elite swimmers following creatine supplementation (9 g/day for 5 days). In terms of swimming, Papadimitriou reviewed the literature surrounding the efficacy of several ergogenic supplements, including creatine [74]. Finally, creatine has also been proposed as a potential ergogenic aid for other total body exercises with similar physiological demands, such as mixed-martial arts; however, limited evidence is currently available.

### 3.4. Endurance Performance

Although creatine supplementation has been quite effective in its ability to function as an ergogenic aid for anaerobic exercise, much less evidence and discussion exists surroundings its ability to benefit endurance performance [75]. When considering creatine’s role from an energetic perspective, this is not surprising, as exercise bouts greater than 2 to 3 min in duration rely predominately on the oxidative system for the synthesis of ATP [76]. Currently, the efficacy of creatine supplementation appears to be more limited for endurance sports, with the magnitude of the benefit being dependent on the duration of the event, as well as the mode of exercise. As exercise duration increases, the ergogenic potential of creatine is diminished [49]. A vast majority of studies show that creatine supplementation has no appreciable effect on maximal oxygen consumption (i.e., VO_2_Max or VO_2_Peak) [7,58,77,78,79,80,81,82], submaximal oxygen consumption [83,84,85], or time trial performance [82,86,87,88,89,90]. A few studies demonstrated improvements in time-to-exhaustion [91,92], but a majority reported no effect [77,82,93,94].

Interestingly, a few research studies have reported small yet significant improvements in other endurance performance variables, such as blood lactate concentration at a given workload and ventilatory/lactate threshold following creatine supplementation [77,95,96]. For example, Chwalbinska-Monteta et al. [95] had elite male rowers perform an incremental exercise to exhaustion on a rowing ergometer before and after consuming 20 g/day of creatine or a placebo for 5 days. Significant reductions in blood lactate at submaximal intensities, as well as increases in lactate threshold, were reported. No differences between groups were found in maximal power or maximal blood lactate concentration after the all-out exercise. In addition to this study, a pair of studies conducted in the same laboratory [77,97] used recreationally active males completing 4 weeks of high-intensity interval training while supplementing with either 10 g per day of creatine citrate or a placebo. During an incremental cycle ergometry test, significant improvements in critical power (+6.7%) and ventilatory threshold (+6%) were observed for the creatine group compared to the placebo group. No significant differences were recorded in VO_2_Peak, time to exhaustion, or total work accomplished. A 2019 study by Fenandez-Landa and colleagues [96] investigated the effects of 10 weeks of creatine on rowing performance. An incremental test to exhaustion was performed by each subject on a rowing ergometer before and after both the creatine and placebo treatments. Measurements included power at anaerobic threshold (WAT), 4 mmol (W4), and 8 mmol (W8) of blood lactate concentration. Power outputs at 8 mmol blood lactate concentration in the creatine-supplemented group increased after eight weeks, whereas no changes were observed in the placebo group, in addition to an approximate 6%–7% increase in absolute power output, which was significantly greater than the absolute power output in the placebo group.

In these studies, endurance performance variables were only slightly improved after creatine supplementation and therefore should be interpreted with caution when considering the practical benefits of using creatine for endurance performance. In addition, the mode of exercise may increase the likelihood of finding ergogenic benefits from creatine. For example, the majority of support for creatine use in endurance exercise has been observed using either an indoor rowing ergometer [95,96] or a cycle ergometer [77,97]. By contrast, the performance of endurance activities such as running, swimming, and soccer involves the propulsion of the body, which may be adversely affected by the gains in body mass typically seen with creatine supplementation, a concept also discussed previously with activities such as jumping. It is quite possible that increases in body mass may counteract any beneficial effects of creatine by increasing the energy demands of exercise during these propulsive/anti-gravity activities [98]. Finally, and as mentioned in previous sections, Mielgo-Ayuso et al. [69] conducted a systematic review and meta-analysis of the effects of creatine supplementation in soccer players. Although 90% of the energy used in soccer match play comes from aerobic metabolism, results indicated that creatine did not improve any aspect of aerobic performance.

Other outcomes of creatine supplementation have been purported to impact endurance training and performance. For example, studies have demonstrated that adding creatine to carbohydrates [44] or carbohydrates + protein supplementation [99] may help promote greater glycogen storage. In many endurance activities, the intensity and duration of training and competition cause drastic reductions in hepatic and intramuscular glycogen levels [1], thus added glycogen storage resulting from adding creatine to carbohydrate and carbohydrates + protein feeding is viewed as an essential benefit. Additionally, the volume and nature of endurance training can invoke noticeable muscle damage. Cooke and colleagues [100] have reported that creatine supplementation may reduce circulating levels of muscle damage markers and help to more quickly restore the ability of the damaged muscle to produce force, whereas other studies have also highlighted a distinct myoprotective role for creatine [101]. Beyond these findings, Santos et al. [102] previously reported that creatine supplementation (5 g/dose four times per day for 5 days) in a group of runners who were monitored for 24 h after completing a 30-km running race experienced reduced levels of soreness, muscle damage, and inflammation. Finally, athletes participating in endurance activities must deal with high ambient temperatures and humidity, which can compromise thermoregulation and subsequently performance. In this respect, studies are available that have documented creatine’s ability to hyper-hydrate the cell and enhance tolerance to heat [103,104]. In considering these findings, athletes may experience a reduction in the risk of heat-related injuries when training or competing in these conditions [3,4,103,104,105]. To summarize, limited research is available that has directly examined the ability of creatine supplementation to impact endurance exercise. Indeed, more work is needed to explore creatine’s potential to impact performance in these types of activities directly. Although some preliminary work suggests that creatine may not directly support endurance performance, other studies have provided many areas of evidence where creatine can provide support and aid the training and performance of these types of athletes (See Table 6).

## 4. Recovery

Increases in intramuscular levels of creatine phosphate secondary to creatine supplementation increase the supply of a robust, energetic substrate that can be used to resynthesize ATP. In this capacity, creatine supplementation can help increase and maintain the delivery of ATP to working muscles, allowing for an increased ability to perform work, resulting in the widespread display of ergogenic outcomes commonly reported in the literature [3,9,19,22,106]. Aside from overt improvements in the performance of single bouts of maximal efforts, creatine is able to augment performance across multiple sets of performance and subsequently demonstrates a role in enhancing recovery. The term recovery is often contextual in nature and typically pertains to either physiological, subjective, or performance-based parameters. In this respect, creatine appears to positively influence recovery in regard to physical performance following bouts of intense activity, and has been shown to enhance recovery during bouts of intermittent activity, sustaining maximal performance across multiple bouts of exercise. In addition, creatine supplementation may also reduce the post-exercise inflammatory response, thereby attenuating markers of muscle damage and soreness in the hours and days following bouts of exercise-induced muscle damage. Finally, creatine may have efficacy as a therapeutic intervention following an injury or during periods of limb immobilization.

### 4.1. Augmented Recovery Following Exercise

#### Augmented Recovery during Intermittent Activities

Creatine supplementation has consistently demonstrated the ability to augment recoverability between bouts of intermittent activity, such as repeated Wingate tests, high-intensity interval training, sprint cycling, sprinting, intermittent team sports, and certain resistance training protocols. As mentioned earlier, oral creatine supplementation is an effective means to increase the intramuscular PCr content by 10%–40% [6], a key component of the ATP-PCr energy system, which is known to contribute a high percentage of ATP yield during high-intensity bouts of activity lasting 0–30 s [7]. For these reasons, it is no surprise that creatine supplementation has been shown to improve intermittent high-intensity exercise performance [3,107]. In this respect, heightened physical performance is thought to be due to a higher initial intramuscular PCr content at the beginning of the exercise and to increased PCr resynthesis during recovery periods between intermittent anaerobic bouts. As such, PCr availability is increased throughout the completion of successive bouts, which subsequently functions to attenuate declines in power output and other indices of fatigue. Moreover, activities characterized by a high force output are more reliant on type II muscle fibers, which have a higher PCr content than the more oxidative type I fibers, and subsequently are favored during bouts of activity at or near maximal effort, with a high degree of force output [108]. Several studies involving many different types of athletes (e.g., cycling, running, swimming, hockey, handball, etc.) supplementing with creatine have documented improvements in intermittent high-intensity exercise capacity [7,42,47,60,73,78,109,110,111,112,113,114,115]. For example, Birch et al. [42] were one of the first to note improvements in high-intensity cycling performance (30 s) during the first two of three maximal effort bouts following five days of creatine supplementation (4 × 5 g/day). Kreider et al. [22] reported greater improvements in total work completed during a 12 × 6 s maximal-effort sprint protocol, particularly during sprints one to five, after 28 days of creatine supplementation in collegiate football players compared to a placebo. Using repeated maximal swimming efforts, Grindstaff and investigators [73] reported significantly faster swim times in their first race. The entire distance covered over swimming was set in 18 male and female junior competitive swimmers after supplementing with either creatine or a placebo for nine days during training. Jones et al. [60] examined skating performance in 16 elite ice-hockey players after having them consume 5 g of creatine monohydrate or placebo (glucose) four times per day for 5 days, after which a maintenance dose of 5 g per day for ten weeks was administered. Subjects completed six timed 80-m skating sprints, with each sprint being initiated every 30 s with a split time taken after 47 m. Mean on-ice sprint skating performance was significantly improved after 10 days, and this ergogenic outcome was sustained through ten weeks of supplementation when compared to baseline performance. Similarly, Mujika et al. [61] reported that 6 days of creatine supplementation (20 g/day) improved repeated sprint performance (6 × 15 m sprints with 30 s recovery) in 17 highly trained male soccer players. Other improvements in intermittent running performance have also been reported following periods of creatine supplementation. For example, Aaserud et al. [116] observed improvements in sprint times during successive (8×) 40-m sprint tests following 15 g of creatine per day over 5 days in well-trained handball players. Similarly, Deminice et al. [117] observed improvements in mean and peak power during a running-based anaerobic sprint test (RAST) consisting of six 35-m sprints following a 7-day creatine supplementation period (0.3 g/kg/day) in under 20 male soccer players.

Some contradictory findings have also been observed following creatine supplementation, whereby no improvements in performance were documented. For example, Green and colleagues [118] supplemented 19 active men with either a placebo or 20 g of creatine per day for 6 days and had them complete three upper-body and three lower-body Wingate tests. No changes in mean power or peak power production were observed for either type of test. However, the magnitude in the observed decrease in power was greater following placebo use but was lesser with creatine supplementation, suggesting that a better attainment of power may have occurred. In addition, Similar outcomes were reported by Ahmun [119] and Deutekom [120], who also failed to observe any ability of creatine loading at a dosage of 20 g/day for 5 and 6 days, respectively, to enhance repeated sprint cycling performance. Similar to repeat cycling sprint performance, Ahmum [119] reported that acute creatine supplementation did not positively impact sprint running performance when compared to a placebo, an outcome that was also reported by Glaister et al. [67]. In summary, several studies are available which have reported on the ability of creatine to positively impact repeated sprint exercise performance. In general, these studies consistently demonstrate an improvement in power production and performance times across multiple bouts of exercise. These findings are not universal, as a limited number of studies have indicated that creatine may not impact performance. Researchers are encouraged to consider the nature of the exercise and training being completed by the study participants, as well as the dosing regimen, when fully evaluating creatine’s potential. Finally, in nearly all of these instances in which an ergogenic outcome was not identified, creatine supplementation, at worst, was able to maintain exercise performance.

### 4.2. Loss of Force Production, Muscle Damage, Soreness, and Inflammation

Preliminary evidence indicates that creatine supplementation may improve recovery from bouts of intensive exercise and subsequently improve physical performance, particularly when a high degree of exercise-induced muscle damage may have occurred. More specifically, and in addition to its recovery of force production potential, it is also possible that creatine supplementation may help to attenuate muscle damage and soreness following damaging bouts of exercise; however, the specific mechanisms underpinning this protective effect have yet to be fully elucidated. For example, Cooke et al. [100] reported greater isokinetic (10% higher) and isometric (21% higher) knee extension strength in a group that supplemented with creatine (0.3 g/kg body weight × 5 days) following a bout of eccentric-only repetitions using 120% of the subjects’ 1RM on leg press, leg extension, and leg flexion exercise machines. The authors noted attenuated creatine kinase levels (a common marker of muscle damage) at 48, 72, and 96 h, and 7 days, following a bout of eccentric exercise in the creatine supplementation group. In a similar study, Rosene et al. [121] reported a higher level of maximal isometric force production in the days following a bout of eccentric leg extensions, a type of exercise known to instigate muscle damage, in those supplementing with creatine after a maintenance dosing protocol, but not after an acute loading dose protocol. A similar attenuation of post-exercise muscle damage was noted in a cohort of Ironman triathletes who completed a creatine (or placebo) loading protocol (20 g/day for 5 days) prior to an Ironman competition [122]. Those supplementing with creatine experienced blunted increases in creatine kinase, lactate dehydrogenase, and aldolase post-competition. The Ironman competitors also experienced an attenuation of glutamic oxaloacetic acid transaminase and glutamic pyruvic acid transaminase following the race, which indicates a dampened inflammatory response. Interestingly, preliminary evidence is also available to suggest that creatine supplementation (20 g/day for 5 days) may attenuate post-exercise increases in markers of muscle damage and soreness, while mitigating reductions in the range of motion above that which is normally present in instances of the “repeated bout effect” during resistance training activities [123]. For more pronounced benefits during resistance training programs, longer periods of creatine supplementation may be required to attenuate post-exercise muscle damage following bouts of resistance-based exercise. For example, Wang et al. [124] indicated that a 4-week period of creatine supplementation (20 g/day for 6 days followed by 2 g/day for 22 days) in conjunction with a complex training regimen reduced the post-exercise increase in creatine kinase compared to a placebo group. Furthermore, a review by Kin et al. [125] summarized the role of creatine supplementation in exercise-induced muscle damage and concluded that creatine might help to prevent muscle damage and facilitate recovery following high-intensity exercise. However, contradictory findings have been observed following bouts of resistance-based exercise, predominantly following shorter periods of creatine supplementation. For example, Rawson et al. [106] had subjects complete 50 maximal eccentric contractions of the elbow flexors, preceded by a creatine (or placebo) loading protocol of 20 g/day for 5 days. Both groups experienced significant losses in maximal isometric force production and range of motion, while experiencing increases in arm circumference, soreness, creatine kinase, and lactate dehydrogenase. These acute physiological responses were similar between groups, indicating that the standard creatine loading protocol used exhibited a limited potential to attenuate markers of muscle damage and soreness following the bout of resistance exercise inducing muscle damage. Similar findings have also been reported in which creatine supplementation did not appear to attenuate markers of muscle damage, soreness, strength deficits, or fatigue following exercise [121,126,127,128,129].

Various intracellular mechanisms have been discussed and proposed that outline the potential anti-oxidant and protective properties of creatine, as first proposed by Lawler et al. in the early 2000s [130]. Following this proposed model, researchers examined if creatine exerted a natural ability to act as an antioxidant against aqueous radical and reactive species ions, using an in vitro model. Shortly thereafter, Sestili et al. [131] observed creatine’s ability to exert mild antioxidant activity in living cells due to creatine acting as a scavenger of reactive oxygen and nitrogen species. In this respect, Santos et al. [102] observed reductions in markers of cell damage and inflammation in runners following a 30 km race who completed a creatine loading protocol prior to the race (20 g/day for 5 days) compared to a control group. Importantly, this work was one of the first investigations to demonstrate the ability of creatine to attenuate the inflammatory response, following an endurance event that resulted in a high degree of muscle damage and pro-inflammatory signaling mechanisms. Using a repeated sprint protocol, Deminice et al. [117] reported the inhibition of TNF-α and C-reactive protein (common inflammatory markers), but not markers of oxidative stress following a 7-day creatine loading protocol (0.3 g/kg/day). As such, it appears that creatine may be more beneficial for endurance activities regarding its ability to reduce indices of post-exercise muscle damage and soreness, in addition to attenuating pro-inflammatory signaling cascades. Although potential exists for creatine supplementation to favorably influence post-exercise indices of muscle damage, soreness, and muscle function, more work is needed to identify the specific types of exercise models and sports activities that may benefit the most and how an acute attenuation of post-exercise muscle damage influences training adaptations over time. Table 7 provides a summary of selected studies examining the effects of creatine supplementation on outcomes relating to muscle damage, soreness, inflammation, and recovery.

### 4.3. Immobilization and Muscle Dysfunction

Periods of limb immobilization and muscle disuse appear to alter the metabolic functioning of skeletal muscle tissue through the downregulation of metabolic pathways, enzyme activity, and organelle function [132,133]. Furthermore, muscle disuse elicits mitochondrial-mediated apoptosis, which is thought to contribute further to muscle atrophy, while concomitantly creating additional metabolic perturbations within the cell [132,134]. Previous research has identified reductions in intracellular phosphagen and glycogen content following muscle disuse [135]. More recently, it was discovered that periods of muscle disuse also resulted in alterations in intracellular phosphagen and creatine transporter content in human skeletal muscle, demonstrating how the cell adapts to the aforementioned metabolic disruptions in cellular energy turnover [136]. These alterations in intracellular phosphate and transporter protein content, coupled with mitochondrial-mediated apoptosis, highlight a rationale for the potential therapeutic benefit of creatine supplementation to attenuate reductions in phosphagen levels associated with muscle disuse. Supplementation may also concomitantly increase the capacity of the ATP-PCr energy system to resynthesize ATP, leading to higher rates of energy turnover during tissue recovery.

Because of the greater phosphagen-mediated re-synthesis of ATP following creatine supplementation, users are likely able to achieve a greater volume load during rehabilitative activities, which may have implications in minimizing muscle atrophy and promoting other favorable adaptations [49]. However, other plausible mechanisms have been proposed for how creatine may confer anabolic properties [137,138]. For example, it has been proposed that increased intracellular osmolarity from augmented creatine storage may cause cell swelling and the concomitant stimulation of anabolic signaling pathways, independently of exercise [138]. Unfortunately, studies assessing the intrinsic capacity of creatine to stimulate hypertrophic protein remodeling are scant. Among the few studies that have been conducted assessing the utility of creatine to ameliorate disuse-mediated muscle atrophy, results are mixed in their findings and primary outcomes. One of the earlier works [139] demonstrated that creatine had no effect on the change in quadriceps cross-sectional area after two weeks of cast immobilization. However, creatine augmented the regeneration of whole muscle and fiber-type-specific cross-sectional area after a 10-week rehabilitation program following the cast immobilization. Mechanistically, the authors also reported that creatine supplementation elevated the myogenic transcription factors myogenin and myogenic factor 4 (Mrf4) [139]. Other work has demonstrated that short-term (5-day) creatine supplementation can upregulate insulin like growth factor-1 (IGF-1) mRNA at rest and augment the phosphorylation of eukaryotic translation initiation factor 4E-binding protein 1 (4E-BP1) 24 h after an acute bout of resistance exercise [140]. Both IGF-1 and 4E-BP1 have the potential to stimulate protein anabolism through PI3K-Akt-mTOR signaling, thereby positively influencing the recovery of the injured tissue [141]. Beyond its putative anabolic effects, creatine supplementation may have anti-catabolic effects as well [142]. However, these findings are not ubiquitous. Although short term oral creatine supplementation was found to attenuate the loss in muscle mass and strength following a period of upper arm immobilization in healthy young men, [143] these same benefits have not been observed following periods of lower limb immobilization [144], knee arthroplasty [145], or anterior cruciate ligament reconstruction [146]. Cumulatively, although these data suggest the potential therapeutic usage of creatine to aid in recovery, more work is needed in this area, particularly regarding whether any potential synergistic benefits may exist when creatine is combined with other nutrients or rehabilitative strategies post-injury.

## 5. Other Considerations

Due to the popularity associated with creatine supplementation since the first published reports in the early 1990s, a number of other questions have been evaluated and considered regarding its efficacy. For example, the majority of the published literature on creatine has been completed using male athletes, leading to much less information being available on how creatine supplementation impacts females. Previous work has highlighted gender-specific differences in creatine production and turnover, which lays the foundation for gender considerations for creatine [4]. In regard to research involving exercise performance in females, Vandenberghe et al. [39] reported that creatine supplementation increases intramuscular PCr levels, muscle mass, and strength when compared to those females who took a placebo. Other research by Hamilton [147] showed improvements in upper-body exercise capacity, and Tarnopolsky showed improvements in high-intensity exercise performance [148], whereas Kambis et al. [149] reported improvements in knee extension muscle performance. Similarly, excellent potential exists for creatine to support the health and function of older populations. Although nearly all of the original research on creatine used young, athletic populations, research in the past 10–15 years has highlighted creatine’s ability to increase the ability to perform daily living activities, to delay fatigue, and to improve muscle mass in older populations [150,151,152,153,154,155,156,157,158].

Overwhelmingly, the majority of research that has examined the potential of creatine to impact exercise performance has been conducted using the monohydrate version. Although several other forms of creatine have been proposed and marketed as alternatives, none have been shown to offer benefits above and beyond those seen with monohydrate. In this respect, a number of studies have been completed comparing various alternative forms of creatine, and the interested reader is directed to the following papers: [3,4,5,30,159,160,161,162,163]. In this respect, one must also realize that several studies have sought to examine the impact of combining creatine with other ingredients, such as beta-alanine [164,165], beta-hydroxy-beta-methylbutyrate (HMB) [96,166,167,168,169,170,171], glutamine [72], sodium bicarbonate [47], carbohydrates [22,44,99,172,173], and protein [22,59,99] to examine the potential for any synergistic outcomes. Furthermore, the interested reader is encouraged to read the critical review on this topic by Jäger et al. [159].

The level of creatine uptake is a key consideration, as it relates to the potential for health and performance outcomes. In this respect, one of the key considerations that has been identified in the literature is the presence of ‘responders’ and ‘nonresponders’. This concept was discussed in a 1999 review by Demant and Rhodes, in which they summarized the available literature and highlighted the fact that identical supplementation regimens could lead to increases in intramuscular PCr levels, whereas the same regimen may cause limited to no changes in other people following a similar supplementation regimen [20]. To illustrate this point, Kilduff et al. [174] identified subjects in their study as responders and nonresponders based on the magnitude of change seen in intramuscular PCr. When examined together, peak force was not changed due to supplementation, but when evaluated separately, the responders significantly increased their peak force production after creatine supplementation. Later, Syrotuik et al. [175] completed an analysis aiming to build a physiological profile of responders and nonresponders. In terms of creatine uptake, a commonly discussed factor that may dictate the extent to which intramuscular PCr levels change in response to creatine supplementation is the level of vegetarianism observed by the individual. In this respect, it is well known that meat-containing foods and products contain the highest amounts of creatine, and thus people who refrain from eating meats tend to consume the lowest amounts of creatine in their diet. Several papers are available that have examined the impact of creatine supplementation on people who follow various types of vegetarianism, with initial reports showing that vegetarians do, indeed, have lower levels of intramuscular PCr [176,177]. Shomrat and colleagues [178] were some of the first to examine this question and concluded that creatine supplementation equally impacted the ability of vegetarian and non-vegetarian individuals to generate peak power after an identical regimen of supplementation. Furthermore, additional studies by Watt [179] and Lukaszuk [180] highlighted the fact that creatine supplementation in vegetarian people can impact intramuscular and plasma levels of creatine in a similar fashion. Furthering this aim, reviews by Venderley and Kaviani concluded that creatine supplementation could be an effective strategy for vegetarian individuals to increase their intramuscular levels of PCr, a key factor that may impact an individual’s ability to perform high-intensity exercise [176,177]. Finally, interested readers are encouraged to review the following articles by Antonio [4], as well as the International Society of Sports Nutrition’s position on creatine [3].

## 6. Conclusions

Augmenting intramuscular creatine stores either by creatine loading or daily supplementation over several days leads to increased concentrations of intramuscular creatine and PCr. Increases in these substrates are associated with an attenuation of ATP degradation, heightened ATP production, and an increase in energetic output during activities involving intermittent, high-intensity, short bouts of exercise. Additionally, creatine supplementation shows promise in facilitating recovery following exercise-induced muscle damage and potentially as an aid during post-injury rehabilitation. Based on the current literature, the following can be deduced involving creatine supplementation and its ergogenic potential:

1. Creatine supplementation is safe during short- and long-term intervals for healthy males and females, as well as in younger and older individuals.
2. Creatine supplementation, ingested at 0.3 g/kg/day for 3–5 consecutive days or 20 g/day for 5–7 successive days, has been shown to quickly increase intramuscular creatine, yielding immediate ergogenic benefits. Correspondingly, a regimen of 3–5 g/day over 4 weeks increased creatine stores, augmented muscle performance, mitigated recovery factors, and resulted in muscle accretion.
3. Creatine supplementation intermixed with carbohydrates or carbohydrates and protein appears to be efficacious in increasing intramuscular creatine retention, although the additional benefits in terms of performance outcomes appear to be nebulous.
4. Creatine supplementation appears to provide an ergogenic effect when assessing isolated or individual bouts of peak or maximal force production.
5. Creatine supplementation facilitates more significant improvements in strength and FFM.
6. Creatine supplementation provides benefits during single and repeated sprints and may increase agility and jumping performance.
7. Creatine supplementation appears to provide ergogenic benefits to aerobic endurance bouts with positive physiological adaptations.
8. Creatine supplementation may enhance recovery from intense exercise and possibly provide synergistic benefits during the post-injury rehabilitation period.
9. Creatine supplementation provides positive benefits to both males and females, athletes and recreational fitness enthusiasts, as well as younger and older individuals.
10. Creatine supplementation provides more significant augmentations of intramuscular creatine in vegans than omnivores, due to lower initial levels of creatine stores, with both groups receiving comparable ergogenic benefits.

## Figures and Tables

**Table 1:** Potential ergogenic benefits of creatine supplementation.

• Increased single and repetitive sprint performance
• Increased work performed during sets of maximal effort muscle contractions
• Increased muscle mass and strength adaptations during training
• Enhanced glycogen synthesis
• Increased anaerobic threshold
• Possible enhancement of aerobic capacity via greater shuttling of ATP from mitochondria
• Increased work capacity
• Enhanced recovery
• Greater training tolerance

Adopted from Kreider et al. 2017 [3].

**Table 2:** Examples of sports and activities in which performance may be enhanced by creatine supplementation.

*Increased PCr*
• Track sprints: 60–200 m
• Swim sprints: 50 m
• Pursuit cycling
*Increased PCr Resynthesis*
• Basketball
• Field hockey
• America Football
• Ice hockey
• Lacrosse
• Volleyball
*Reduced Muscle Acidosis*
• Downhill skiing
• Water Sports (e.g., Rowing, Canoeing, Kayaking, Stand-Up Paddling)
• Swim events: 100, 200 m
• Track events: 400, 800 m
• Combat Sports (e.g., MMA, Wrestling, Boxing, etc.)
*Oxidative Metabolism*
• Basketball
• Soccer
• Team handball
• Tennis
• Volleyball
• Interval Training in Endurance Athletes
*Increased Body Mass/Muscle Mass*
• American Football
• Bodybuilding
• Combat Sports (e.g., MMA, Wrestling, Boxing, etc.)
• Powerlifting
• Rugby
• Track/Field events (Shot put; Javelin; Discus; Hammer Throw)
• Olympic Weightlifting

Adopted from Kreider et al. 2017 [3].

**Table 3:** Summary of selected studies examining the acute effects of creatine monohydrate on strength performance.

Author and Year	Subjects	Duration	Dosing Protocol	Primary Variables	Results	Adverse Events
Peak Torque/Force Production						
Greenhaff et al. 1993	12 healthy males	5 days	20/d for 5 days	isokinetic performance during repeated intervals	↑ muscle torque ↓ plasma ammonia ↔ BLa	None reported
Casey et al. 1996	9 active males	5 days	20 g/day for 5 days	cycling sprint performance	↑ total work	None reported
Gilliam et al. 2000	23 healthy males	5 days	20 g/day for 5 days	isokinetic performance of quadriceps	↔ muscle torque	None reported
Rossouw et al. 2000	13 trained powerlifters	5 days	9 g/day for 5 days	isokinetic knee extension	↑ peak torque ↑ average power, ↑ total work ↑ work output ↑ deadlift volume	None reported
Kilduff et al. 2002	32 trained males	5 days	20 g/day for 5 days	isometric bench press	↑ peak force ↑ total force	None reported
Strength Performance						
Birch et al. 1994	14 healthy males	5 days	20 g/day for 5 days	isokinetic cycling performance during repeated intervals	↑ mean power output ↑ peak power output ↑ total power output ↓ plasma ammonia ↔ BLa	None reported
Barnett et al.1996	17 active males	4 days	280 mg/kg for 4 days	10 s sprints on a cycle ergometer	↔ mean power output ↔ peak power output ↔ blood pH ↔ BLa	None reported
Strength and Performance Outcomes						
Edwards et al. 2000	21 active males	6 days	20 g/d for 6 days	anaerobic intervals on a treadmill	↔ speed test performance ↔ BLa ↓ plasma ammonia	None reported
Rockwell et al. 2001	16 trained males	4 days	20 g/day for 4 days	repeated cycling sprints	↔ total work ↔ maximal power ↔ work capacity	None reported
Volek et al. 2001	10 healthy males	7 days	0.3 g/kg for 7 days	repeated cycling sprints cardiovascular and thermoregulatory responses	↔ cardiovascular or thermoregulatory responses ↑ peak power ↑ mean power	None reported
Finn et al. 2001	16 male triathletes	5 days	20 g/day for 5 days	repeated cycling sprints	↔ peak power ↔ fatigue index ↔ mean power	None reported
Ziegenfuss et al. 2002	20 (10 male, 10 female) power athletes	3 days	0.35 g/kg of fat-free mass for 3 days	repeated cycling sprints	↑ peak power ↑ total work	None reported
Yquel et al. 2002	9 healthy males	6 days	20 g/day for 6 days	repeated dynamic plantar flexion muscle pH	↑ mean power ↔ muscle pH	None reported
Delecluse et al. 2003	12 (7 male, 5 female) competitive sprinters	7 days	0.35 g/day for 7 days	single 40-m sprint repeated 40-m sprints	↔ single 40-m sprint ↔ repeated 40-m sprints	None reported
Kocak et al. 2003	20 elite male wrestlers	5 days	20 g/day for 5 days	average and peak power during wingate anaerobic power test	↑ average power ↑ peak power	None reported
Selsby et al. 2004	31 trained males	10 days	2.5 g/day for 10 days	bench press strength bench press endurance	↔ bench press strength ↔ bench press endurance	None reported
Zuniga et al. 2012	22 active males	7 days	20 g/day for 7 days	wingate cycling test lower body strength upper body strength	↑ mean power output ↔ leg extension ↔ bench press	None reported
del Favero et al. 2012	34 untrained males	10 days	20 g/day for 10 days	lower body strength upper body strength	↑ bench press ↑ squat	None reported
Wang et al. 2017	17 high school canoeists	6 days	20 g/day for 6 days	upper body strength overhead medicine ball throw Post-activation potentiation	↑ upper body strength ↔ overhead medicine ball throw ↔ post-activation potentiation	None reported

BLa = blood lactate; CR or no identifier = creatine monohydrate; ↓ indicates decrease; ↑ indicates increase; ↔ indicates no difference.

**Table 4:** Summary of selected studies examining the long-term (>2 weeks) effects on strength performance.

Author and Year	Subjects	Duration	Dosing Protocol	Primary Variables	Results	Adverse Events
Peak Torque/Force Production						
Stevenson et al. 2001	18 (17 males, 1 female) trained subjects	9 weeks	20 g/day for 7 days 5 g/day 8 weeks	maximal torque on isokinetic dynamometer quadriceps cross-sectional area	↔ maximal torque ↑ cross-sectional area	None reported
Chrusch et al. 2001	30 healthy older males	12 weeks	0.03 g/kg/day for 5 days 0.07 g/kg/day for 11 weeks	lean tissue bench press strength leg press strength knee extension muscle endurance average power	↑ lean tissue ↔ bench press ↑ leg press ↑ knee torque ↑ muscle endurance ↑ average power	None reported
Strength						
Vandenberghe et al.1997	19 healthy female subjects	11 weeks	5 g/day for 4 days 2.5 g/day for 10 weeks	arm flexion on isokinetic dynamometer upper and lower body muscle strength body composition	↑ arm torque ↑ leg press, leg extension, squat ↔ bench press, leg curl, shoulder press ↑ lean muscle mass	None reported
Kelly et al. 1998	18 male powerlifters	26 days	20 g/day for 5 days 5 g/day for 21 days	bench press strength bench press endurance total body mass	↑ bench press strength ↑ bench press endurance ↑ body mass	None reported
Volek et al. 1999	19 trained males	12 weeks	25 g/day for 7 days 5 g/day for 11 weeks	body mass fat-free mass bench press strength squat strength quadriceps cross-sectional area	↔ body mass ↑ fat-free mass ↑ bench press ↑ squat ↑ quadriceps cross-sectional area	None reported
Becque et al. 2000	23 trained males	6 weeks	20 g/day for 5 days 2 g/day for days 6–42	upper body strength body composition	↑ arm flexor strength ↑ body mass ↔ body fat ↑ fat-free mass ↑ upper arm muscle area	None reported
Brenner et al. 2000	20 female college lacrosse players	5 weeks	20 g/day for 7 days 2 g/day for days 8–32	body composition bench press strength knee extension strength knee extension endurance BLa	↔ body composition ↑ bench press strength ↔ knee extension strength ↔ knee extension endurance ↔ BLa	None reported
Larson-Meyer et al. 2000	14 female college soccer players	13 weeks	15 g/day for 5 days 5 g/day 5 days/week for 12 weeks	bench press strength squat strength vertical jump body composition	↑ bench press strength ↑ squat strength ↔ vertical jump ↑ body mass ↑ lean mass ↔ body fat	1 subject reported nausea
Bemben et al. 2001	25 male college football players	9 weeks	20 g/day for 5 days 5 g/day for 8 weeks	bench press strength squat strength power clean strength Wingate cycling test isokinetic strength body composition	↑ bench press strength ↑ squat strength ↑ power clean strength ↑ anaerobic power, capacity and % decrement ↑ peak torque knee flexion ↔ peak torque knee extension ↑ lean body mass ↔ body fat	None reported
Burke et al. 2001	47 active male subjects	21 days	7.7 g/day for 21 days	bench press on a isokinetic dynamometer	↑ peak force ↑ peak power ↑ time to fatigue	None reported
Chrusch et al. 2001	30 healthy older males	12 weeks	0.03 g/kg/day for 5 days 0.07 g/kg/day for 11 weeks	lean tissue bench press strength leg press strength knee extension muscle endurance average power	↑ lean tissue ↔ bench press ↑ leg press ↑ knee torque ↑ muscle endurance ↑ average power	None reported
Wilder et al. 2001	25 male college football players	10 weeks	3 g/day for 10 week or 20 g/day for 7 days, then 5 g/day for rest of the study	squat strength body composition	↔ squat strength ↔ lean body mass ↔ fat mass	None reported
Burke et al. 2003	49 (20 male, 29 female) active subjects	8 weeks	0.25 g/kg lean tissue/day for 7 days 0.0625 g/kg lean tissue/day for 49 days	bench press strength leg press strength isokinetic endurance quadriceps cross-sectional area body composition	↑ bench press ↔ leg press ↑ total work ↑ body mass ↑ lean body mass ↑ cross-sectional area	None reported
Ferguson et al. 2006	26 trained females	10 weeks	0.3 g/kg for 7 days 0.03 g/kg for 9 weeks	bench press strength leg press strength body composition	↔ bench press ↔ leg press ↔ total mass ↔ lean body mass ↔ fat mass	None reported
Kerksick et al. 2009	24 trained males	4 weeks	20 g/day for 5 days 5 g/day for 23 days	bench press strength leg press strength isokinetic knee extension Wingate cycling test body composition	↑ bench press ↑ leg press ↔ peak torque ↔ peak power ↑ lean body mass ↑ fat-free mass	None reported
Camic et al. 2010	22 untrained males	28 days	5 g/day for 28 days	bench press strength leg extension strength Wingate cycling test	↑ bench press ↔ leg extension ↔ mean power ↔ peak power	None reported
Hummer et al. 2019	22 (16 males, 6 females) active subjects	6 weeks	4 g/day for 6 weeks	bench press strength bench press endurance squat strength squat endurance	↑ bench press strength ↑ bench press endurance ↑ squat strength ↔ squat endurance	None reported
Strength and Performance Outcomes						
Kreider et al. 1998	25 college football players	28 days	15.75 g/day for 28 days	total work during sprints on a cycle ergometer bench press volume total volume	↑ total work ↑ bench press volume ↑ total volume	None reported
Stone et al. 1999	42 college football players	5 weeks	0.22 g/kg/day for 5 weeks	bench press strength squat strength countermovement vertical jump static vertical jump body composition	↑ bench press strength ↔ squat strength ↔ countermovement vertical jump ↔ static vertical jump ↑ body mass ↑ lean body mass	None reported
Chilibeck, et al. 2007	19 male union rugby players	8 weeks	0.7 g/kg/day for 8 weeks	bench press endurance leg press endurance body composition	↔ bench press repetitions ↔ leg press repetitions ↑ when combining bench press and leg press scores ↔ total body mass ↔ lean tissue mass ↔ fat mass	None reported

BLa = blood lactate; CR or no identifier = creatine monohydrate; ↓ indicates decrease; ↑ indicates increase; ↔ indicates no difference.

**Table 5:** Summary of selected studies examining the effects of creatine supplementation on sport performance.

Author Year	Subjects	Duration	Dosing Protocol	Primary Variables	Results	Adverse Events
Grindstaff et al. 1997	18 (7 male, 11 female) junior competitive swimmers	9 days	21 g/day for 9 days	100-m sprint performance arm ergometer performance	↑ sprint swimming performance	None reported
Kreider et al. 1998	25 college football players	28 days	15.75 g/day for 28 days	total work during sprints on a cycle ergometer bench press volume total volume	↑ total work ↑ bench press volume ↑ total volume	None reported
Noonan et al. 1998	39 college football players	9 weeks	20 g/day for 5 days 100 or 300 mg/kg/fat-free mass for 8 weeks	bench press 40-yard dash % body fat fat-free mass vertical jump	↑ bench press ↑ 40-yard dash ↔ % body fat ↔ fat-free mass ↔ vertical jump	None reported
Peyrebrune et al. 1998	14 male college swimmers	5 days	9 g/day for 5 days	single 50-m sprint time repetitive 50-m sprint time	↔ single 50 m sprint time ↑ repetitive 50 m sprint time	None reported
Stout et al. 1999	24 college football players	8 weeks	21 g/day for 5 days 10 g/day thereafter	vertical jump 100-yard dash bench press strength	↑ vertical jump ↑ 100-yard dash ↑ bench press strength	None reported
Jones et al. 1999	8 elite ice hockey players	11 weeks	20 g/day for 5 days 5 g/day for 10 weeks	5 × 15 s skating sprints 6 timed 80-m skating sprints	↑ 5 × 15 s skating sprints ↑ 6 timed 80 m skating sprints	None reported
Kirksey et al. 1999	36 (16 male, 20 female) track and field athletes	6 weeks	0.3 g/kg/day	countermovement vertical jump power and total work during sprints on a cycle ergometer	↑ countermovement vertical jump ↑ peak power ↑ total work on cycle ergometer	None reported
Kreider et al. 1999	61 college football players	12 weeks	20–25 g/day	Bench press strength Bench press endurance Body composition	↑ bench press strength ↑ bench press endurance ↑ body mass ↑ soft tissue lean mass	None reported
Mujika et al. 2000	17 trained soccer players	10 weeks	20 g/day for 5 days 5 g/day for 9 weeks	countermovement jump repeated sprint ability	↔ countermovement jump ↑ repeated sprint ability	None reported
Haff et al. 2000	36 (16 male, 20 female) track and field athletes	6 weeks	0.3 g/kg/day	countermovement vertical jump	↑ countermovement vertical jump	None reported
Skare et al. 2001	18 male competitive sprinters	5 days	20 g/day for 5 days	100-m sprint time total sprint time (6 × 60 m)	↑ 100 m sprint time ↑ total sprint time	None reported
Romer et al. 2001	9 competitive squash players	5 days	0.075 g/kg 4 times for 5 days	single sprint repetitive sprint performance	↔ single sprint ↑ repetitive sprint performance	None reported
Izquierdo et al. 2002	19 male handball players	5 days	20 g/day for 5 days	countermovement vertical jump repetitive sprint performance	↑ countermovement vertical jump ↑ 6 × 15 m sprints	None reported
Cox et al. 2002	12 elite female soccer players	6 days	20 g/day for 6 days	agility kick drill test agility race test repetitive sprint performance BLa	↔ kick drill test ↑ agility run ↑ repetitive sprint performance ↓ BLa	None reported
Lehmkul et al. 2003	29 (17 male, 12 female) track and field athletes	8 weeks	0.3 g/kg/day for 7 days 0.03 g/kg/day for 7 weeks.	average and peak power during repeated sprints on a cycle ergometer	↔ static vertical jump ↔ countermovement vertical jump ↔ average power ↔ peak power	None reported
Delecluse et al. 2003	12 (7 male, 5 female competitive sprinters	7 days	0.35 g/day for 7 days	single 40-m sprint repeated 40-m sprints	↔ single 40 m sprint ↔ repeated 40 m sprints	None reported
Kocak et al. 2003	20 elite male wrestlers	5 days	20 g/day for 5 days	average and peak power during Wingate anaerobic power test	↑ average power ↑ peak power	None reported
Ostojic et al. 2004	20 young male soccer players	7 days	30 g/day for 7 days	dribbling test sprint power countermovement jump	↑ dribbling test ↑ sprint power ↑ countermovement vertical jump	None reported
Pluim et al. 2006	36 competitive tennis players	32 days	0.3 g/day for 6 days 0.03 g/day for 28 days	serve velocity groundstroke velocity repetitive sprints	↔ serve velocity ↔ groundstroke velocity ↔ repetitive sprints	None reported
Glaister et al. 2006	42 active males	5 days	20 g/day for 5 days	repetitive sprint performance	↔ repetitive sprint performance	None reported
Lamontagne-Lacasse et al. 2011	12 elite male volleyball players	28 days	20 g/day in days 1–4 10 g/day in days 5–6 5 g/day in days 7–28	repeated block jump spike jump	↔ repeated block jump ↔ spike jump	None reported
Ramierz-Campillo et al. 2016	30 amateur female soccer players	6 weeks	20 g/day for 7 days 5 g/day for 5 weeks	jump test repeated sprinting directional change	↑ jump test ↑ repeated sprinting ↔ directional change	None reported

BLa = blood lactate; CR or no identifier = creatine monohydrate; BLa = blood lactate; ↓ indicates decrease; ↑ indicates increase; ↔ indicates no difference.

**Table 6:** Summary of selected studies examining the effects of creatine supplementation on endurance performance.

Author-Year	Subjects	Duration	Dosing Protocol	Primary Variables	Results	Adverse Events
Rossiter et al. 1996	38 (28 male, 10 female) competitive rowers	5 days	0.25 g/kg/day for 5 days	time trial performance during rowing ergometry	↓ 2.3 s in 1000-m times	None reported
McNaughton et al. 1998	16 elite male paddlers	5 days	20 g/day for 5 days	total work, peak power, BLa during rowing ergometry	↑ in total work during 90–300 s of rowing ergometry performance	None reported
Miura et al. 1999	8 healthy males	5 days	20 g/day for 5 days	critical power test during cycle ergometry	↔ critical power ↑ anaerobic work capacity	None reported
Rico-Sanz et al. 2000	14 elite male cyclists	5 days	20 g/day for 5 days	oxygen consumption, time to exhaustion, BLa during maximal cycle ergometry	↔ VO2 max ↑ time to exhaustion ↔ BLa	None reported
Syrotuik el al. 2001	22 (12 male, 10 female) competitive rowers	6 weeks	0.3 g/kg/day for 5 days 0.03 g/kg/day for 5 weeks	time trial performance during rowing ergometry	↔ in 2000-m rowing times	None reported
Jones et al. 2002	9 active males	5 days	20 g/day for 5 days	VO2 kinetics during moderate and heavy submaximal cycle exercise	↔ VO2 kinetics ↓ VO2 during heavy cycling exercise	None reported
Chwalbinska-Moneta 2003	16 elite male rowers	5 days	20 g/day for 5 days	maximal power output, time to exhaustion, Bla during rowing ergometry	↔ maximal power output ↑ time to exhaustion ↔ BLa	None reported
Graef et al. 2009	43 active males	30 days	10 g/day for 20 days; only on training days (5 × week)	oxygen consumption, time to exhaustion, VT, total work, during maximal cycle ergometry	↔ VO2 peak ↑ time to exhaustion ↑ ventilatory threshold ↔ Total work	None reported
Kendall et al. 2009	43 active males	30 days	10 g/day for 20 days; only on training days (5 × week)	critical power and anaerobic work capacity during cycle ergometry	↑ Critical power ↔ Anaerobic work capacity	None reported
Hickner et al. 2010	12 endurance-trained males	28 days	3 g/day for 28 days	VO2peak, submaximal VO2, RER, Bla, 10 s sprints at 110% VO2peak during simulated cycling road race	↔ VO2peak ↓ submaximal VO2 ↔ RER ↔ Bla, ↔ 10-s sprints at 110% VO2peak	2 subjects reported muscle cramping at rest following supplementation
De Andrade Nemezio et al. 2015	24 male amateur cyclists	5 days	20 g/day for 5 days	time trial performance total O2 uptake, BLa during maximal cycle ergometry	↔ 1000 m time ↓ total O2 uptake ↔ BLa	None reported
Forbes et al. 2017	17 active females	28 days	0.3 g/kg/day for 5 days 0.1 g/kg/day for 23 days	VO2peak, VT, peak workload, time trial performance during cycle ergometry	↔ VO2peak ↔ VT ↔ 2000-m time ↔ peak workload	None reported
Fernandez-Landa et al. 2020	28 elite male rowers	10 weeks	0.04 g/kg/day for 10 weeks + 3 g HMB/day for 10 weeks	power output at AT, 4 mmol, 8 mmol Bla during rowing ergometry	↑ power at AT for creatine-HMB and HMB only group ↑ power at 4 mmol BLa for creatine-HMB group ↑ power at 8 mmol BLa for creatine only, HMB only, and creatine-HMB groups	None reported

BLa = blood lactate; CR or no identifier = creatine monohydrate; RER = respiratory exchange ratio; ↓ indicates decrease; ↑ indicates increase; ↔ indicates no difference.

**Table 7:** Summary of selected studies examining the effects of creatine supplementation on indices of muscle damage, inflammation, and recovery.

Author Year	Subjects	Duration	Dosing Protocol	Primary Variables	Results	Adverse Events
Oopik et al. 2002	5 well-trained male wrestlers	17 hours	30 g (7.5 g/serving) + 320 g glucose (80 g/serving) over 4 doses	isokinetic performance blood glucose blood lactate plasma ammonia plasma urea body mass	↑ submaximal work ↔ blood glucose ↔ Bla ↓ plasma ammonia ↔ plasma urea ↔ body mass	None reported
Hespel et al. 2001	22 (13 males, 9 females)	10 weeks	20 g/day for 3 weeks (immobilization) 15 g/day for 3 weeks (10–8) 5 g/day for 7 weeks (7–1).	quadriceps cross-sectional area (CSA) knee extension isometric force	↑ CSA ↑ knee torque ↑ isometric force	None reported
Tyler et al. 2004	60 ACL reconstruction patients (33 males, 27 females)	6 months	20 g/day for 7 days 5 g/day for 12 weeks	knee extension knee flexion hip flexion hip abduction hip adduction single leg hop	↑ knee outcome measures comparing to baseline	None reported
Rawson et al. 2007	22 trained males	10 days	0.3 g/kg/day for 5 days 0.03 g/kg/day for 5 days	maximal strength range of Motion muscle Soreness blood lactate	↔ strength ↔ ROM ↔ soreness ↔ Bla	None reported
Cooke et al. 2009	14 untrained males	20 days	0.3 g/kg/day + glucose (80 g/day) for 5 days 0.1 g/kg/day + glucose (0.4 g/day) for 14 days	isokinetic force isometric force	↑ isokinetic force ↑ isometric force	None reported
Rosene et al. 2009	20 healthy males	30 days	20 g/day for 7 days 6 g/day for 23 days	isometric force knee range of motion muscle soreness creatine kinase blood lactate	↑ isometric force	None reported
Johnston et al. 2009	7 healthy males	30 days	Maltodextrin 20 g/day for 7 days (Day 1–7) 20 g/day for 7 days (Day 15–21)	fat free mass elbow flexor strength and endurance elbow extensor strength & endurance	↑ lean tissue ↑ muscular strength ↑ muscular endurance	None reported
McKinnon et al. 2012	27 (15 male, 12 female) untrained subjects	10 days	40 g/day + CHO 40 g/day for 5 days 5 g/day + CHO 5 g/day for 5 days.	muscle force loss rate of recovery muscle soreness	↔ force loss ↔ rate of recovery ↔ muscle soreness	None reported
Boychuk et al. 2016	14 healthy males	48 hours	0.3 d/kg	maximal voluntary contraction muscle thickness electromyography muscle soreness	↔ strength ↔ EMG activation ↔ muscle soreness	None reported
Backx et al. 2017	30 healthy males	12 days	20 g/day for 5 days 5 g/day for 7 days	quadriceps cross-sectional area (CSA) leg 1 RM knee extensions	↔ CSA ↔ 1 RM	None reported

1 RM = one repetition maximum; CHO = carbohydrates; CR or no identifier = creatine monohydrate; BLa = blood lactate; ↓ indicates decrease; ↑ indicates increase; ↔ indicates no difference.

## Data Availability

Data sharing not applicable as no new data were analyzed as part of this review paper.
